# Bacterial metabolite-directed synthesis of biogenic TiO_2_–Zn nanocomposites: characterization and multifunctional biomedical evaluation

**DOI:** 10.1039/d6ra00881j

**Published:** 2026-04-27

**Authors:** Ibrahim M. Ibrahim, Hanadi A. Alahmadi, Anes A. Al-Sharqi, Nidal Mohammed Zabermawi, Mohammed Alsieni, Dareen Alyousfi, Faten A. S. Alsulaimany, Dalal Alfawaz, Issam Alshami, Zinab Alatawi, Ahmed Eid Alharbi, Ahmed Ghareeb

**Affiliations:** a Department of Clinical Pharmacology, Faculty of Medicine, King Abdulaziz University Jeddah 21589 Saudi Arabia imibrahim1@kau.edu.sa malsieni@kau.edu.sa dalfawaz@kau.edu.sa; b College of Health Science and Nursing, Alrayan National Colleges Madinah 42541 Saudi Arabia ha.alahmadi@amc.edu.sa; c Photonics Unit, Institute of Laser for Postgraduate Studies, University of Baghdad Al-Jadiriah, P.O. Box 47314 Baghdad Iraq anes@ilps.uobaghdad.edu.iq; d Sustainable Agriculture Production Research Group, Department of Biological Sciences, King Abdulaziz University Jeddah 21589 Saudi Arabia nzabermawi@kau.edu.sa; e Department of Clinical Biochemistry, Faculty of Medicine, King Abdulaziz University 21589 Jeddah Saudi Arabia dalyousfi@kau.edu.sa; f Institute of Genomic Medicine Sciences, King Abdulaziz University Jeddah Saudi Arabia; g Department of Biological Sciences, Faculty of Science, King Abdulaziz University Jeddah 21589 Saudi Arabia Faalsulaimany@kau.edu.sa; h Department of Basic Medical Sciences, College of Medicine, Taibah University Madinah 42353 Saudi Arabia ishami@taibahu.edu.sa; i Department of Family and Community Medicine, Faculty of Medicine, University of Tabuk Tabuk 47512 Saudi Arabia zalatawi@ut.edu.sa; j Department of Medical Laboratory, College of Applied Medical Sciences in Yanbu, Taibah University Yanbu Governorate Saudi Arabia aeharbi@taibahu.edu.sa; k Botany and Microbiology Department, Faculty of Science, Suez Canal University Ismailia 41522 Egypt aghareeb@science.suez.edu.eg

## Abstract

This study synthesized TiO_2_–Zn nanocomposites using metabolites from Red Sea-isolated *Bacillus tequilensis* MYG163 and evaluated their multifunctional therapeutic potential. Chronic diseases such as diabetes, infections, and inflammation share overlapping pathological mechanisms that single-target therapies cannot adequately manage. XRD analysis confirmed the presence of anatase TiO_2_ and wurtzite ZnO phases with crystallite sizes of 23.1 and 23.7 nm, respectively, in the TiO_2_–Zn nanocomposite. TEM analysis revealed spherical particles with sizes ranging from 8 to 15 nm, while DLS analysis indicated a hydrodynamic diameter of 87.3 nm and a polydispersity index of 0.232. EDX analysis indicated the presence of Ti (32.1 wt%), Zn (29.2 wt%), and O (33.8 wt%) and a zeta potential of −34.5 mV, confirming colloidal stability. Hemolysis remained below 0.7% across all concentrations tested (50–1000 µg mL^−1^), confirming blood compatibility, essential for biomedical applications. DPPH and ABTS radical scavenging assays yielded IC_50_ values of 11.97 and 7.65 µg mL^−1^, respectively. Anti-inflammatory testing demonstrated preferential COX-2 inhibition (IC_50_ = 14.13 µg mL^−1^) over COX-1 (IC_50_ = 25.91 µg mL^−1^), representing a therapeutically favorable selectivity profile that minimizes gastrointestinal side effects associated with nonselective inhibition as well as prevents BSA denaturation (IC_50_ = 2.78 µg mL^−1^). Antimicrobial assays showed inhibition zones of 35 ± 0.4 mm (*B. subtilis*), 33 ± 0.5 mm (*C. albicans*), 26 ± 0.3 mm (*S. typhi*), 25 ± 1.0 mm (*K. pneumoniae*), 25 ± 0.6 mm (*F. oxysporum*), and 22 ± 0.4 mm (MRSA), with activities matching or exceeding those of reference antibiotics and antifungals against several tested organisms. Antidiabetic screening revealed the inhibition of α-amylase and α-glucosidase with IC_50_ values of 12.98 and 9.34 µg mL^−1^, respectively. Marine bacterial metabolites functioned as reducing and stabilizing agents, yielding nanocomposites with multitarget therapeutic properties spanning oxidative, inflammatory, microbial, and metabolic pathways.

## Abstract 2

Biogenic TiO_2_–Zn nanocomposites from Red Sea *Bacillus tequilensis* MYG163 form an anatase–wurtzite heterojunction (8–15 nm) with antioxidant, anti-inflammatory, antimicrobial, antidiabetic, and hemocompatibility activities for multitarget therapy.

## Graphical abstract

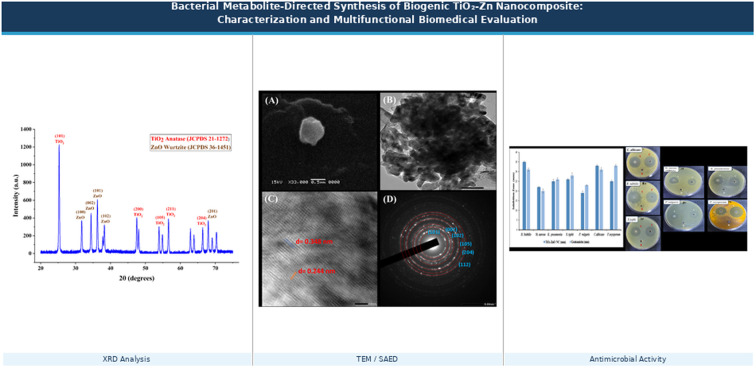

## Introduction

Standard therapies are often insufficient for treating diabetes, inflammation, infections, and oxidative stress, as these conditions arise from interlinked biological pathways that single-target drugs cannot fix.^1^ Diabetes worsens when high glucose causes oxidative damage in the retina and kidneys, but conventional treatments only lower blood sugar without stopping the underlying tissue destruction.^2^ Patients often discontinue their medications because of weight gain, stomach issues, and high costs.^3^ Natural compounds that work on multiple pathways handle both cellular stress and inflammation better than conventional drugs.^4^ Traditional medicines provide rapid relief and have established safety profiles, but they do not address the underlying causes of chronic diseases.^5^

Metal oxide nanoparticles show promise for medical applications due to their unique biological activities.^6^ TiO_2_ is a widely studied semiconductor metal oxide characterized by chemical stability, low toxicity, and broad-spectrum biological activity,^7^ making it attractive for drug delivery, antimicrobial, and tissue engineering applications.^8^ Similarly, ZnO is also recognized for its potent antimicrobial properties, which are attributed to zinc-ion release and reactive oxygen species generation, alongside established antidiabetic and anti-inflammatory activities.^9^ TiO_2_ degrades organic contaminants *via* photocatalysis under UV irradiation, achieving degradation efficiencies of up to 98%. It kills multiple pathogen types while remaining compatible with living tissues, making it useful for drug transport and tissue repair.^10^ ZnO fights microorganisms by releasing metal ions and generating reactive oxygen species that damage bacterial and viral structures.^11^ Combining TiO_2_ with ZnO yields better results than those obtained using either oxide separately. The heterojunction between these materials improves electron transfer and reduces charge recombination, boosting both photocatalytic efficiency and contaminant removal rates.^12^ Combining TiO_2_ with ZnO produces a heterojunction binary system with enhanced biological performance compared to that of either oxide alone, which is attributed to improved charge carrier separation at the interface between the two materials.^13^ This combination substantially increases antimicrobial strength, and studies have reported that the inhibitory concentration drops by a factor of 8 compared to single-component systems.^14^ The heterostructure keeps reactive species active for a longer duration, which helps eliminate pathogens and neutralize free radicals more effectively.

Conventional chemical and physical routes for producing metallic nanoparticles rely on toxic reagents that harm people and the environment, consume large amounts of energy, and generate considerable waste. High production costs also prevent their use at the industrial scale.^15^ Biological methods using bacteria, plants, and fungi address these problems by providing cleaner and cheaper alternatives. Microbes release metabolites that reduce metal ions to their zero-valent states while coating the particle surfaces to stop aggregation and improve stability.^16^*Bacillus* species are particularly well-suited for nanoparticle fabrication, producing extracellular enzymes and proteins that direct particle nucleation and growth, yielding materials with controlled dimensions suitable for medical applications.^17^ Research shows that *Bacillus* strains create particles with precise sizes and shapes that fit the requirements for medical, agricultural, and environmental applications.^18^ This biological route bypasses the hazards and ecological damage of standard methods while delivering stable nanomaterials at lower costs.^18^

Research on TiO_2_–Zn nanocomposites produced *via* bacterial synthesis reveals significant gaps, particularly for strains isolated from Red Sea ecosystems. Studies typically examine single properties rather than testing antioxidant, anti-inflammatory, antimicrobial, antidiabetic, and wound-healing activities within one experimental framework.^19–21^ Red Sea microbial communities represent an underexplored reservoir of metabolically active strains capable of directing the formation of nanoparticles with distinct physicochemical properties.^22^ Comprehensive biological evaluations that measure safety and therapeutic effectiveness across multiple disease targets are largely absent. This fragmented approach prevents understanding whether bacterially synthesized TiO_2_–Zn NCs can address diverse medical needs simultaneously.^23^

This study synthesized TiO_2_–Zn NCs using metabolites from Red Sea-isolated *Bacillus tequilensis* MYG163 and examined their biomedical properties. Characterizations including FT-IR spectroscopy, XRD, TEM, EDX, DLS, and zeta potential analyses were used to establish the particle structure, composition, and surface characteristics. Biological testing measured hemolytic response, radical neutralization *via* DPPH and ABTS assays, modulation of inflammation *via* COX-1 and COX-2 inhibition, prevention of BSA denaturation, pathogen suppression against bacteria and fungi, and glucose regulation by targeting α-amylase and α-glucosidase enzymes.

This work investigated whether bacterium-mediated synthesis could yield nanocomposites with therapeutic values across the biomedical applications investigated.

## Materials and methods

### Bacterial extract preparation and biogenic TiO_2_–Zn NC synthesis

The bacterial strain *Bacillus tequilensis* MYG163 was isolated from the coastal environments of the Red Sea and confirmed taxonomically through 16S rRNA sequencing (GenBank: OR906149). Our previous studies have focused on the extraction of its exopolysaccharide fraction (EPS-R1), characterization of its molecular composition, and evaluation of its biomedical capabilities.^24^ Bacterial strains were grown in marine broth at 37 °C for 72 hours with shaking at 150 rpm to produce the metabolite. The culture was then centrifuged at 8000 rpm for 15 minutes to pellet the cells. The supernatant underwent triple extraction with ethyl acetate (1 : 1 v/v), with each organic layer collected separately. Combined extracts were concentrated by rotary evaporation under vacuum at 40 °C, reconstituted in ethyl acetate, and stored at 4 °C before use for TiO_2_–Zn nanoparticle synthesis.^24^

0.395 g of titanium oxide (anatase, −325 mesh, ≥99%, purchased from Sigma Aldrich, CAS no. 1317-70-0, catalogue no. 248576) was dispersed in 25 mL of dH_2_O and then combined with 25 mL of the bacterial extract. Concurrently, zinc sulphate (20 mM) was dispersed in 25 mL of distilled water and mixed with 25 mL of the bacterial extract. Zn^2+^ ions released from zinc sulphate and Ti^4+^ ions released from titanium oxide underwent bioreduction mediated by the enzymes, proteins, and polysaccharides present in the bacterial extract, affording the biogenic TiO_2_–Zn NC, consistent with previously reported Bacillus-mediated metal oxide synthesis. Each mixture was stirred at 600 rpm for 48 hours. The two solutions were then combined and agitated at 700 rpm for an additional 24 hours. Following centrifugation at 8000 rpm for 10 minutes, the pellet was collected and stored in a sealed microtube for downstream characterization and biomedical assessments.^20^

### Physicochemical characterization of the biogenic TiO_2_–Zn NC

The TiO_2_–Zn NC samples were first heated at 45 °C for 24 hours to remove residual moisture. Following this desiccation step, the dried material was intimately mixed with a KBr powder to create a uniform composite. The resulting mixture was then pressed into a thin disc and examined by Fourier transform infrared (FT-IR) spectroscopy using a Nicolet 6700 instrument (Thermo Fisher Scientific). The spectral region between 400 and 4000 cm^−1^ was investigated to identify and map the vibrational modes associated with the various surface functional groups and chemical bonds present in the nanocomposite.^25^ The crystal structure evaluation of the TiO_2_–Zn NC was performed *via* X-ray diffraction (XRD) employing a PANalytical-X'Pert-Pro-MRD diffractometer equipped with CuKα radiation (*λ* = 1.54 Å).^26^ Diffraction data were collected within the angular range from 10° to 80° (2*θ*) with the instrument operating at 40 kV and 30 mA, providing insights into the lattice ordering and phase composition of the nanocomposite.^27^ The surface morphology of the TiO_2_–Zn NC was examined by scanning electron microscopy using a JEOL JSM-6360LA instrument (Japan) at an accelerating voltage of 15 kV. Samples were mounted on aluminum stubs and sputter-coated with gold prior to imaging.^28^ Structural details were further elucidated by transmission electron microscopy (TEM) using a JEOL JEM-2100 Plus apparatus (Tokyo, Japan) operating at 200 kV, which enabled the direct visualization of the surface topography, particle geometry, and size heterogeneity within the sample. To prepare the sample for TEM analysis, dry powder was dispersed in deionized H_2_O *via* sonication and then transferred to carbon-coated copper grids using the drop-deposition method. After allowing the samples to dry under ambient conditions, high-resolution imaging was performed to characterize the microstructural features.^29^

The quantitative determination of the constituent elements within the TiO_2_–Zn NC was accomplished using energy-dispersive X-ray spectroscopy (EDX) coupled to a JEOL JSM6360LA scanning electron microscope (Japan).^30^ The hydrodynamic diameter and polydispersity of TiO_2_–Zn dispersed in the liquid phase were ascertained *via* dynamic light scattering (DLS) using a Malvern Nano-ZS instrument (Malvern Ltd, UK).^31^ To remove extraneous scattering contributions and noise, stock suspensions of the nanocomposite were prepared by dispersing the material in ultrapure Milli-Q water. Zeta potential measurements, indicative of the electrostatic surface characteristics, were subsequently performed employing the same Malvern Nano-ZS apparatus fitted with a Zeta-sizer module while maintaining identical sample preparation and measurement conditions throughout the investigation.^32^

### Biomedical evaluation of the TiO_2_–Zn NC

#### Hemocompatibility assessment

Erythrocytes were obtained from one of the authors. All experiments were performed in accordance with the Guidelines of the Research Ethics Committee, and experiments were approved by the ethics committee of the Suez Canal University (REC125/2022). Informed consent was obtained from the human participants of this study. Erythrocytes were harvested and subjected to three sequential washes with isotonic saline (150 mM NaCl, Sigma-Aldrich, CAS 7647-14-5) by centrifugation at 2500 rpm in 10-minute intervals. The purified cellular suspension was then reconstituted in phosphate-buffered saline (PBS, Sigma-Aldrich, pH 7.4) to achieve a final cell concentration of 2%. A concentration gradient of TiO_2_–Zn samples ranging from 50 to 1000 µg mL^−1^ was prepared for testing. Deionized water (Milli-Q, Merck) served as the positive control, inducing complete cell lysis (designated as 100% hemolysis), whereas the absence of the nanocomposite material (0 µg mL^−1^) served as the negative control, representing baseline hemolytic activity. Phosphate-buffered saline was retained as the spectrophotometric reference for background subtraction. Each TiO_2_–Zn NC formulation was combined with the RBC suspension at a total reaction volume of 1 mL. The resulting mixtures were incubated at 37 °C for 60 minutes, followed by centrifugation at 2500 rpm for 15 minutes. The resulting supernatants were subsequently analysed spectrophotometrically at 546 nm to quantify the released haemoglobin content.^33^Hemolysis percentage (%) = [(Abs_TiO_2_–Zn_ − Abs_blank_)/Abs_positive control_] × 100

### Antioxidant assessment

#### DPPH assay

A 0.1 mM 2,2-diphenyl-1-picrylhydrazyl (DPPH, MilliporeSigma, USA) solution in ethanol (Univar Solutions, USA) was prepared. 1 mL of this solution was mixed with 3 mL of the TiO_2_–Zn NC suspension (1.9–1000 µg mL^−1^, diluted from ethanol stocks). After shaking, the mixture was allowed to rest for 30 minutes at room temperature, with ascorbic acid (Vivion Inc, USA) serving as the control. The absorbance was measured at 517 nm using a Milton Roy UV-vis spectrophotometer.^34^DPPH scavenging % = [(ascorbic acid_Absorbance_ − TiO_2_–Zn_Absorbance_)/ascorbic acid_Absorbance_] × 100

#### ABTS˙^+^ scavenging assay

The ABTS radical cation was generated by mixing 7 mM 2,2′-azino-bis(3-ethylbenzothiazoline-6-sulfonic acid) (ABTS, Cayman Chemical, USA) with 2.45 mM potassium persulfate (K_2_S_2_O_8_, Junteng Chemical, China) and keeping the mixture in the dark. The TiO_2_–Zn NC sample (0.07 mL) was added to 3 mL of the diluted ABTS˙^+^ solution.^35^ The reaction was allowed to proceed for 6 minutes, and then, the absorbance was measured at 734 nm using UV-vis spectrophotometry. Ascorbic acid served as the ref. 36.ABTS˙^+^ inhibition % = [(ascorbic acid_Absorbance_ − TiO_2_–Zn_absorbance_)/ascorbic acid_Absorbance_] × 100

### Anti-inflammatory assessment of the TiO_2_–Zn NC

#### COX enzyme inhibition assay

The TiO_2_–Zn NC's activity against COX-1 and COX-2 was tested using commercial screening kits (COX-1 catalogue k548 and COX-2 catalogue k547; BioVision, USA). The nanomaterial was dissolved in DMSO (analytical grade, Gaylord Chemical, USA) and tested at concentrations ranging from 1000 to 0.5 µg mL^−1^ in 1-mL reaction mixtures. Celecoxib (Merck, Germany) functioned as the positive control for both assays.^37^COX inhibition % = [(Celecoxib − TiO_2_–Zn NC)/celecoxib] × 100

#### BSA protein denaturation anti-inflammatory assay

50 µL of the TiO_2_–Zn NC preparation was mixed with 450 µL of a 1% aqueous solution of bovine serum albumin (BSA, Sigma-Aldrich/Merck, Germany). Eight dilutions were tested: 1.56, 3.12, 6.25, 12.5, 25, 50, 100, and 200 µg mL^−1^. The pH was brought to 6.3 by adding 1 N HCl dropwise. After 20 minutes at room temperature, the tubes were placed in a 55 °C water bath for 30 minutes. Once removed, they were allowed to reach ambient temperature before the absorbance was measured at 670 nm using a Biosystem 310 plus spectrophotometer. Diclofenac sodium (LGM Pharma, USA) acted as the reference control.^38^% BSA inhibition = (Abs_diclofenac sodium_ − Abs_TiO_2_–Zn NC_/Abs_diclofenac sodium_) × 100

### Antimicrobial testing of biogenic TiO_2_–Zn NC

Agar well diffusion testing was used to assess the antimicrobial performance of the TiO_2_–Zn NC against ATCC reference strains. Gram-positive bacteria included *Bacillus subtilis* ATCC 6633 and methicillin-resistant *Staphylococcus aureus* ATCC 33591 (MRSA). Gram-negative organisms comprised *Klebsiella pneumoniae* ATCC 13883, *Salmonella typhi* ATCC 6539, and *Proteus vulgaris* NCTC 4175/ATCC 13315, all cultured on Mueller–Hinton agar (HiMedia, India, M173). Fungal screening on Sabouraud dextrose agar (HiMedia, India, M063) included *Candida albicans* ATCC 10221 and *Fusarium oxysporum* ATCC 46995. Gentamicin and fluconazole (API manufacturers, China) served as the bacterial and fungal benchmarks, respectively.

Inoculum suspensions derived from broth microdilution were introduced to plates within 15 minutes. Three-directional streaking resulted in the uniform distribution of microbes across the dried agar. Sterile 6-mm cork borers were used to create wells using the aseptic technique. The TiO_2_–Zn NC at 10 µg mL^−1^ in DMSO (100 µL per well) was filled into each opening.^39^

Incubation differed by the microbe type: bacteria grew at 37 °C for 24 h, *Candida* species at 35 °C for 48 h, and *Fusarium* species at 28 °C for 48–72 h. Suppression zones were quantified to the nearest millimetre (mm) where growth ceased markedly.^28^

### *In vitro* bioassay for antidiabetic potential

#### α-Amylase inhibition assay

The inhibitory effect of the TiO_2_–Zn NC on α-amylase (Type VI-B, Sigma-Aldrich, USA, A3176) was quantified using the 3,5-dinitrosalicylic acid (DNS, MilliporeSigma, USA) reduction assay. The TiO_2_–Zn NC underwent evaluation at concentrations ranging from 1.95 to 1000 µg mL^−1^, with acarbose (Bayer AG, Germany) serving as the positive control at matching dose levels. The optical density was measured at 540 nm using a UV-visible Biosystem 310 spectrophotometer.^40^α-Amylase inhibition (%) = [(Abs_acarbose_ − Abs_TiO_2_–Zn_)/Abs_acarbose_] × 100

#### α-Glucosidase inhibition assay

The TiO_2_–Zn NC (1.95–1000 µg mL^−1^) was screened for the suppression of the α-glucosidase (Sigma-Aldrich, USA, G5003) catalytic function. Its inhibitory potency was directly compared with that of acarbose (Bayer AG, Germany), used as the comparative standard. Optical readings were obtained at 405 nm using a Biosystem 310 Plus spectrophotometer, and inhibition percentages were calculated using the standard formula. IC_50_ estimates were derived *via* linear regression analysis by plotting the inhibition percentage against the TiO_2_–Zn NC concentration (1.95–1000 µg mL^−1^).^41^α-Glucosidase inhibition % = [(Abs_acarbose_ − Abs_TiO_2_–Zn_)/Abs_acarbose_] × 100

### Statistical analysis

Statistical tests were performed using SPSS version 29. The Shapiro–Wilk test was used to assess whether the data followed a normal distribution. One-way ANOVA examined differences among experimental groups, with Tukey's HSD posthoc test identifying specific between-group differences.

## Results and discussion

### Physicochemical characteristics of the biogenic TiO_2_–Zn NC

**Fig. 1:**
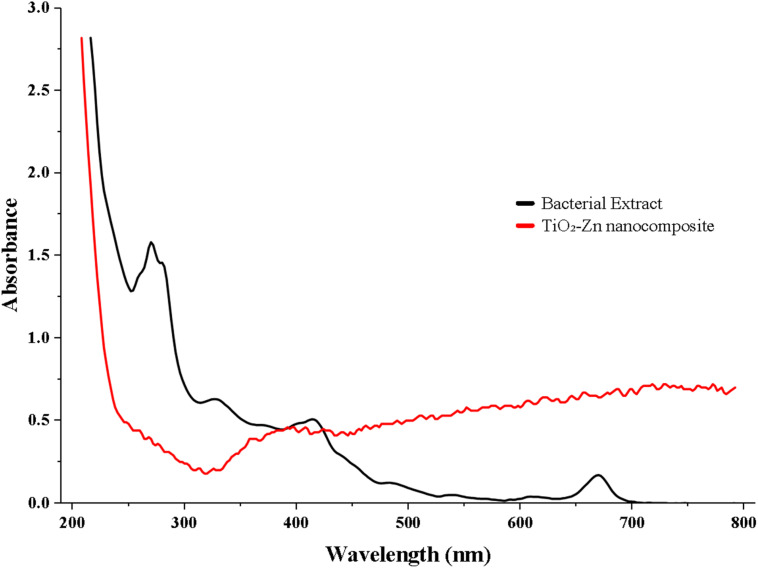
Absorption spectra of the bacterial abstract and the biogenic TiO_2_–Zn NC.

The UV-vis spectra of the bacterial extract and biogenic TiO_2_–Zn NC are presented in Fig. 1. The bacterial extract (black line) showed strong absorption at 214 nm due to protein–peptide bond transitions, with a shoulder at 270–280 nm reflecting aromatic amino acids and a band at 326 nm corresponding to phenolic compounds. The biomolecules responsible for metal-ion reduction and nanoparticle surface capping during synthesis exhibited FT-IR peak shifts at 1645 → 1631 cm^−1^ (amide I), 1543 → 1524 cm^−1^ (amide II), and 1078 → 1066 cm^−1^ (polysaccharide C–O).

The TiO_2_–Zn NC's spectrum (red line) showed a dominant absorption at ∼210 nm, corresponding to TiO_2_ electronic transitions, confirming nanoparticle formation, followed by a sharp decline to a minimum near 280 nm and then a gradual rise through the visible region (400–800 nm). This visible-range tail, absent in pure TiO_2_, indicates bandgap narrowing due to zinc incorporation into the crystal lattice, consistent with the anatase–wurtzite heterojunction confirmed by XRD. The disappearance of the extract's biological absorption bands at 270–280 nm and 326 nm in the NC's spectrum confirms that bacterial metabolites act as reducing agents during synthesis rather than persisting as surface residues.

**Fig. 2:**
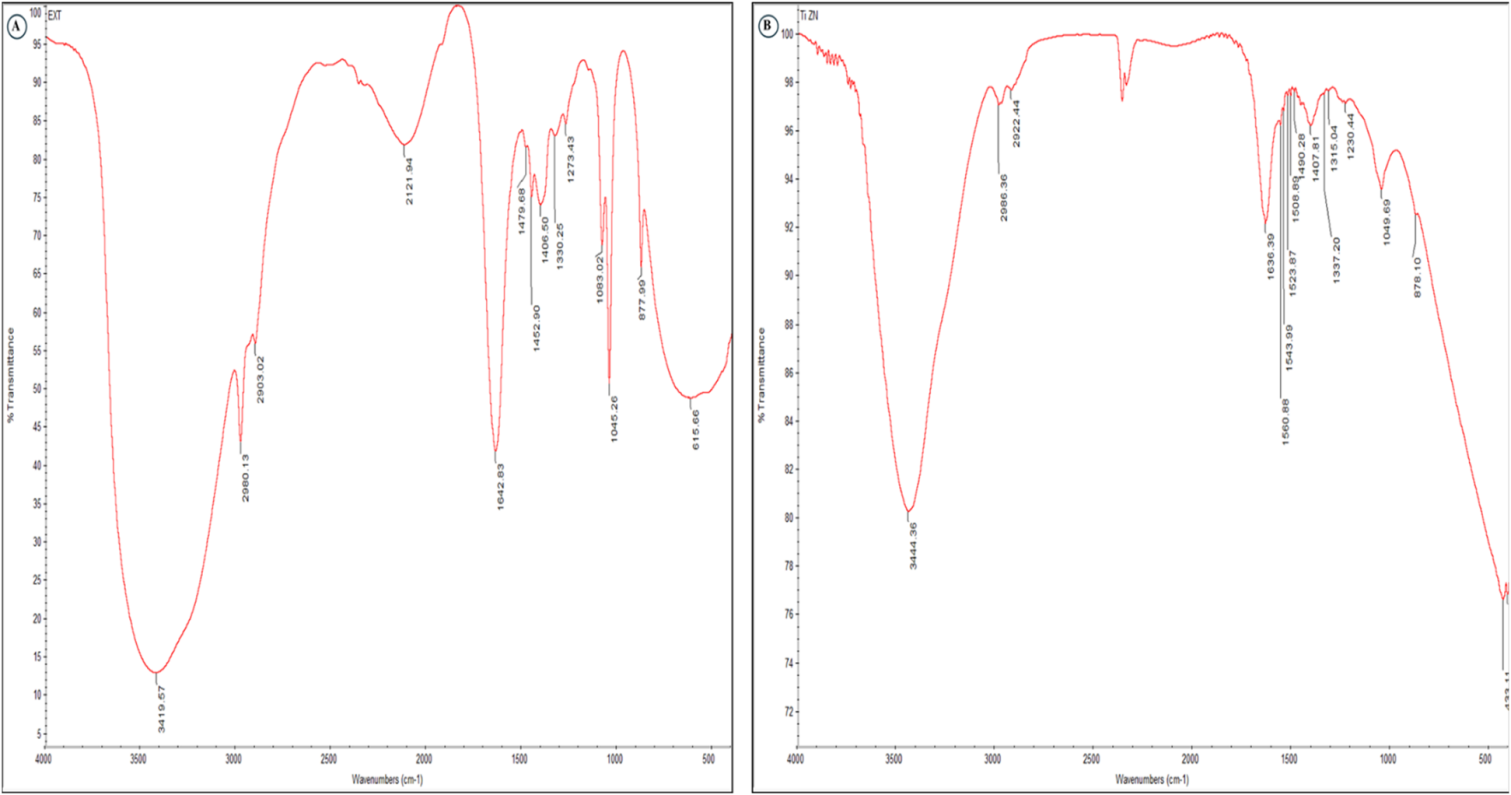
FT-IR spectra of the bacterial extract (A) and TiO_2_–Zn NCs (B) with comparative peak assignments.

The FT-IR spectra revealed substantial structural modification during nanocomposite synthesis. The broad hydroxyl and amine bands near 3400 cm^−1^ shifted to lower frequencies, accompanied by a reduction in intensity, indicating hydrogen bond formation between biomolecules and the growing metal oxide surface. The aliphatic C–H stretching peaks at 2926 and 2855 cm^−1^ underwent minor red-shifts, suggesting an interaction between bacterial metabolites and nanoparticle surfaces without the complete degradation of organic capping agents. Amide I and II bands experienced significant shifts from 1645 to 1631 cm^−1^ and 1543 to 1524 cm^−1^, respectively, confirming protein coordination with metal centers through carbonyl and amine groups (Fig. 2 and Table 1). The carboxylate symmetric stretch moved from 1396 to 1384 cm^−1^, demonstrating the bidentate binding of carboxylic acids to metal ions.

**Table 1:** FT-IR peak assignments and shifts comparing the bacterial extract and TiO_2_–Zn NCs

Bacterial extract (cm^−1^)	TiO_2_–Zn NCs (cm^−1^)	Functional group	Vibrational mode	Peak shift (cm^−1^)	Interpretation
3420	3398	O–H, N–H	Stretching	−22	Hydrogen bonding between biomolecules and the nanoparticle surface
2926	2918	C–H (aliphatic)	Asymmetric stretching	−8	Reduced organic content or structural rearrangement
2855	2848	C–H (aliphatic)	Symmetric stretching	−7	Interaction of alkyl chains with the metal-oxide surface
1645	1631	C=O (amide I)	Stretching	−14	Protein adsorption onto the nanoparticle surface
1543	1524	N–H, C–N (amide II)	Bending, stretching	−19	Coordination of peptide groups with metal ions
1456	1448	C–H	Bending	−8	Conformational changes in organic ligands
1396	1384	COO^−^ (carboxylate)	Symmetric stretching	−12	Carboxyl group binding to Zn^2+^ and Ti^4+^ ions
1243	1231	C–O, P=O	Stretching	−12	Phosphate or polysaccharide interaction with nanoparticles
1078	1066	C–O (polysaccharides)	Stretching	−12	Capping by exopolysaccharide components
876	862	C–H aromatic	Out-of-plane bending	−14	Aromatic compounds involved in the reduction process
—	658	Ti–O	Stretching	New peak	Formation of titanium oxide bonds
—	542	Zn–O	Stretching	New peak	Formation of zinc oxide bonds
—	468	Ti–O–Zn	Bridging vibration	New peak	Heterojunction formation between TiO_2_ and ZnO

The polysaccharide signatures around 1078 cm^−1^ shifted downward, verifying exopolysaccharide involvement in nanoparticle stabilization. Most critically, new absorption bands emerged at 658, 542, and 468 cm^−1^ corresponding to Ti–O, Zn–O, and Ti–O–Zn bridging vibrations, respectively, providing the direct evidence of successful nanocomposite formation and the presence of a heterojunction architecture between the two metal oxides.

**Fig. 3:**
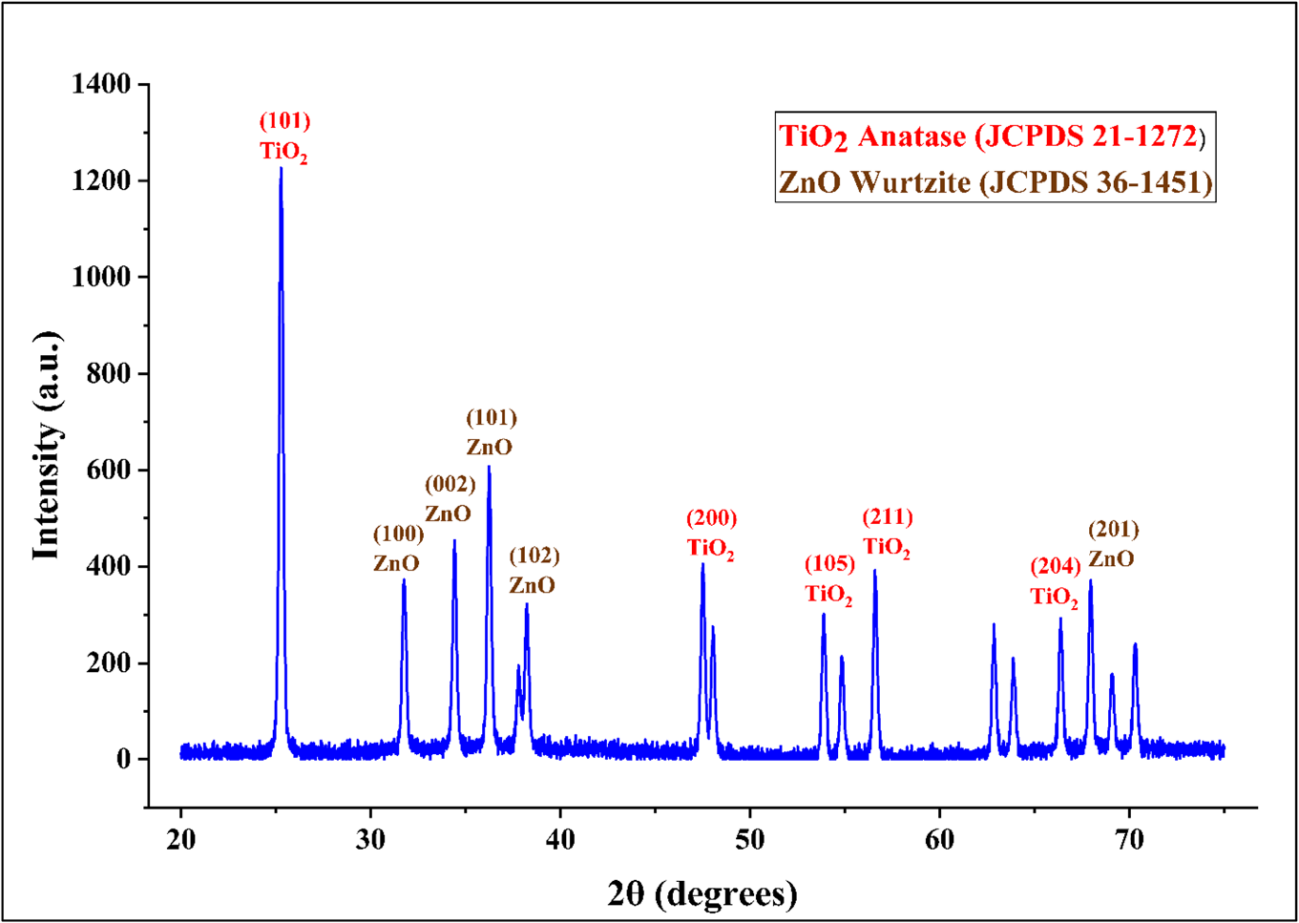
XRD pattern of the TiO_2_–ZnO NC biosynthesized from *Bacillus tequilensis* metabolites, showing the anatase (JCPDS 21-1272) and wurtzite (JCPDS 36-1451) phases.

The biosynthesized TiO_2_–Zn NC derived from *Bacillus tequilensis* MYG163 metabolites exhibited a binary crystalline structure comprising anatase TiO_2_ (tetragonal, space group *I*4_1_/*amd*) and wurtzite ZnO (hexagonal, space group *P*6_3_*mc*), confirmed by matching JCPDS references 21-1272 and 36-1451, respectively. Seventeen distinct diffraction peaks in the 2*θ* range of 20°–75° corresponded to eight TiO_2_ reflections, with the dominant (101) anatase peak at 25.28° (*d* = 3.52 Å and intensity = 1200 a.u.), alongside nine ZnO reflections, including the characteristic (101) wurtzite peak at 36.25° (*d* = 2.48 Å and intensity = 580 a.u.; Fig. 3). Scherrer analysis from the full-width half-maximum (0.353°) yielded crystallite sizes of 23.1 nm for anatase TiO_2_ and 23.7 nm for wurtzite ZnO, indicating nearly uniform nanoscale dimensions.

Phase quantification based on integrated peak intensities revealed approximately equal proportions of TiO_2_ (51.6%) and ZnO (48.4%), establishing a balanced binary oxide system. Sharp, well-resolved diffraction peaks with minimal baseline fluctuations, coupled with an exceptional signal-to-noise ratio (148.5) and peak-to-background ratio (11.95), confirm high crystallinity and phase purity, with no detectable secondary phases or impurities. The absence of preferred-orientation effects and the systematic indexing of all major reflections validate the successful green synthesis of a homogeneous nanocomposite structure suitable for photocatalytic applications.

**Fig. 4:**
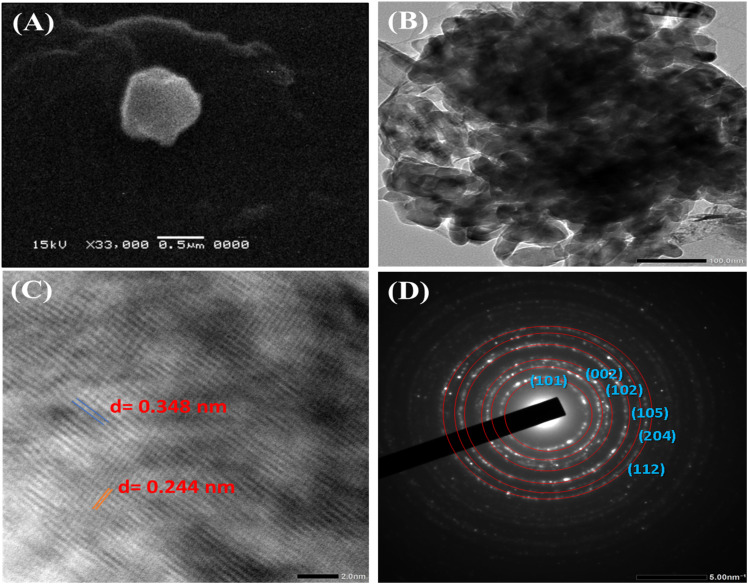
Electron microscopy characterization of the biogenic TiO_2_–Zn nanocomposite: (A) SEM micrograph (330 00×; scale bar = 0.5 µm); (B) TEM image (scale bar = 100 nm); (C) HRTEM lattice fringe image (scale bar = 2 nm); and (D) SAED pattern showing polycrystalline diffraction rings indexed to anatase TiO_2_ and wurtzite ZnO phases.

The SEM micrograph (Fig. 4A) recorded at 330 00× magnification showed a single quasi-spherical particle with a diffuse organic corona surrounding the electron-dense core, a peripheral halo attributable to the bacterial metabolite layer deposited during biogenic synthesis and consistent with the amide I/II band shifts and carboxylate stretch displacement observed in FT-IR spectroscopy.

TEM imaging (Fig. 4B) at the 100-nm scale showed primary crystallite domains assembled into a compact particle, with clearly distinguishable grain boundaries between TiO_2_ and ZnO domains. The granular multidomain texture and the density of these interfaces were consistent with the heterojunction architecture confirmed by HRTEM and the balanced phase proportions of 51.6% TiO_2_ and 48.4% ZnO determined by XRD. HRTEM (Fig. 4C) resolved two coexisting sets of lattice fringes within the same imaged domain, *d* = 0.348 nm, assigned to the (101) plane of anatase TiO_2_ (JCPDS 21-1272), and *d* = 0.244 nm, assigned to the (101) plane of wurtzite ZnO (JCPDS 36-1451, reference *d* = 0.248 nm). Their simultaneous resolution in one region is direct crystallographic proof of a two-phase nanocomposite rather than a physical mixture. The SAED pattern (Fig. 4D) reinforced this conclusion through continuous polycrystalline rings indexed to the (101), (200), and (204) planes of anatase TiO_2_ alongside the (002), (102), and (112) planes of wurtzite ZnO, with random crystallographic orientations confirming the intimate intermixing of both phases. This structural picture is fully consistent with the XRD-determined phase proportions of 51.6% TiO_2_ and 48.4% ZnO, the Scherrer crystallite sizes of 23.1 and 23.7 nm, respectively, the Ti–O–Zn bridging vibration at 468 cm^−1^ in FT-IR spectra, and the EDX quantification of Ti at 32.1 wt% and Zn at 29.2 wt%, collectively establishing the biogenic material as a structurally integrated TiO_2_/ZnO heterojunction nanocomposite.

**Fig. 5:**
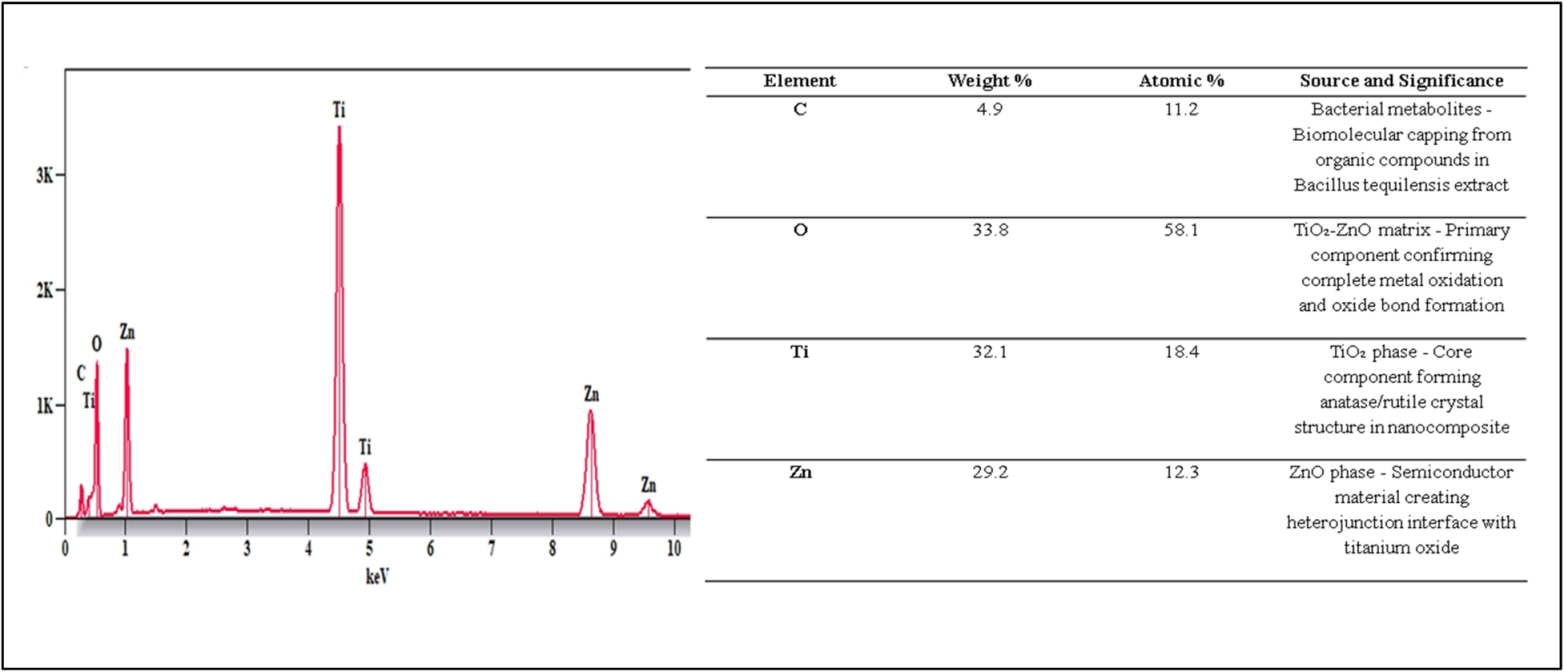
EDX spectrum of the biogenic TiO_2_–Zn nanocomposite and its elemental composition analysis.

EDX analysis verified the elemental composition of the biogenic TiO_2_–Zn NC. The spectrum revealed Ti at 32.1 wt% (18.4 at%), Zn at 29.2 wt% (12.3 at%), and O at 33.8 wt% (58.1 at%), confirming the successful synthesis of the binary metal oxide system (Fig. 5). Carbon, detected at 4.9 wt% (11.2 at%), originated from bacterial metabolites adsorbed on nanoparticle surfaces during biogenic synthesis, consistent with FT-IR spectra observations of organic capping agents.

The atomic percentage of oxygen (58.1%) exceeded the combined metal content, indicating that both titanium and zinc were fully oxidized during nanoparticle formation. The Ti : Zn atomic ratio of approximately 1.5 : 1 suggests the preferential incorporation of titanium into the nanocomposite, which may influence heterojunction properties and charge-carrier dynamics at the metal–oxide interface.

**Fig. 6:**
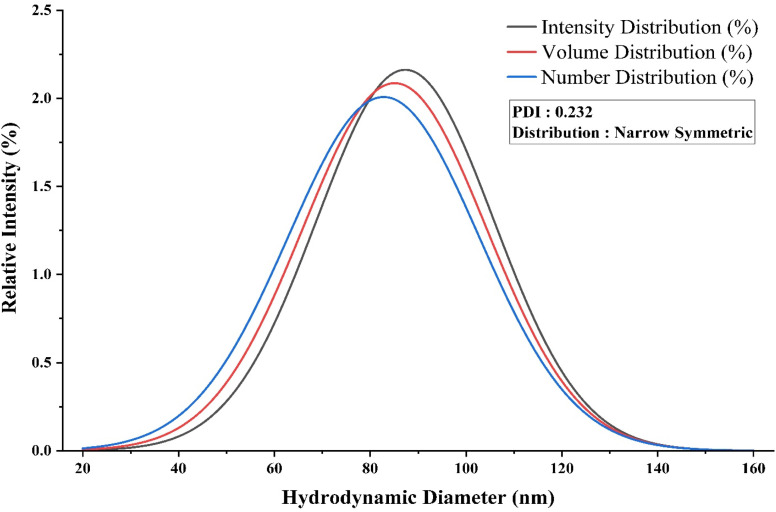
Particle size distribution of the TiO_2_–ZnO NC, showing monodispersed formation with a *Z*-average of 87.3 nm and a PDI of 0.232.

The biosynthesized TiO_2_–ZnO NC exhibited an intensity-weighted *Z*-average diameter of 87.3 nm with a polydispersity index of 0.232, placing it within the monodisperse classification (PDI < 0.3). The particle size distribution displayed a symmetric Gaussian profile across intensity (87.3 nm), volume (85.1 nm), and number (82.8 nm) weighting methods, with minimal deviation between modes (5.4% range, Fig. 6). The narrow distribution width (*σ* = 18.45 nm) and consistent PDI values (0.232–0.258) across all measurement approaches indicate controlled nucleation during biogenic synthesis, with the bacterial metabolites functioning as effective stabilizing agents that prevent aggregation.

The overlapping distribution curves centered around 80–90 nm, lacking secondary peaks or extended tailing, confirm homogeneous particle formation without polydispersed subpopulations. This size regime (80–90 nm) positions the nanocomposite favorably for photocatalytic applications, where surface area scales with reactivity, while the tight distribution ensures reproducible antimicrobial performance by eliminating size-dependent activity variations.

**Fig. 7:**
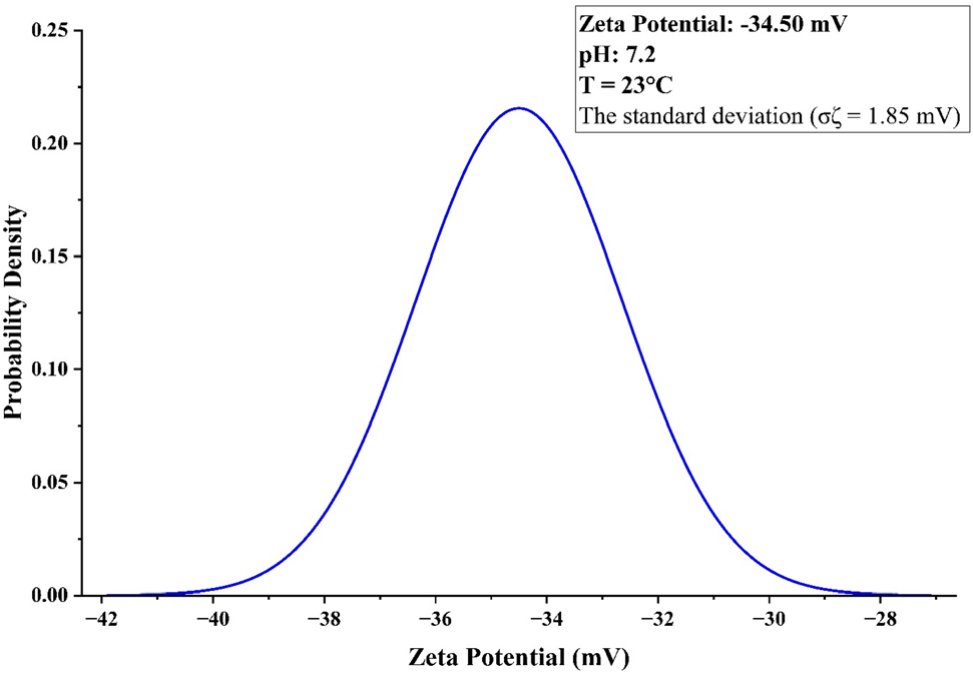
Zeta potential distribution of the TiO_2_–ZnO NC, showing a mean value of −34.5 mV with a narrow deviation (*σζ* = 1.85 mV) at pH 7.2, indicating strong colloidal stability.

The zeta potential of −34.5 mV with a narrow deviation of 1.85 mV demonstrates the strong colloidal stability of the TiO_2_–ZnO NC at pH 7.2, exceeding the ±30 mV threshold required for long-term dispersion stability. The sharp symmetric Gaussian distribution centered at −34.5 mV indicates a uniform surface charge across the particle population, reflecting consistent hydroxyl group deprotonation at near-neutral pH (Fig. 7).

This negative surface potential generates sufficient electrostatic repulsion to prevent aggregation in aqueous media, maintaining the monodisperse character observed in DLS measurements.

TiO_2_–Zn NCs derived from microbial sources exhibited varied morphologies, with shapes and sizes determined by synthesis techniques, reaction conditions, and biological templates employed.^42^ These distinct structural characteristics directly affect their functional performance, particularly in photocatalysis and pathogen inhibition, broadening their potential uses across different fields.^43^ In accordance with our findings, lemon extract yielded spherical TiO_2_–Zn NCs measuring approximately 25 nm in diameter ,^44^ whereas lignin-based methods generated rod-like ZnO structures with sizes between 30 and 70 nm.^45^*Hibiscus rosa-sinensis*, the Chinese hibiscus, facilitated the formation of ellipsoidal ZnO and spherical TiO_2_, while ZnO/Zn_2_TiO_4_ composites exhibited fluffy aggregate-like morphologies forms having dimensions ranging from 18 to 350 nm.^46^ Similarly, the *Trichoderma citrinoviride* extract generated TiO_2_ particles in various forms, such as triangles, pentagons, spheres, and rods, measuring between 10 and 400 nm, with a zeta potential of 29.5 mV.^47^ Hexagonal ZnO NPs produced alongside TiO_2_ measured about 57.87 nm on average and showed strong stability with promising antimicrobial activity.^48^

### Hemolytic activity of the TiO_2_–Zn NC

The hemolytic activity assessment of the TiO_2_–Zn NC demonstrates excellent biocompatibility across all tested concentrations. The results show remarkably low hemolysis percentages ranging from 0.3% to 0.7%, with the highest value of 0.7% observed at a 600 µg mL^−1^ concentration. Even at the maximum tested concentration of 1000 µg mL^−1^, hemolysis remains minimal at 0.3%, well below the 5% threshold typically considered safe for biomedical applications. The absorbance values stay consistently low (0.005 to 0.031) across all concentrations, indicating negligible red blood cell membrane disruption compared to the complete hemolysis control (1.509 ± 0.009, Table 2). These findings position the TiO_2_–Zn NC as a promising candidate for biomedical applications where blood contact is anticipated, such as drug delivery systems or diagnostic agents, because they preserve erythrocyte integrity even at relatively high exposure levels.

**Table 2:** Concentration-dependent hemolysis assessment of the TiO_2_–Zn NC, showing absorbance values and hemolysis percentages

Sample/control	Concentration (µg mL^−1^)	Absorbance mean ± SD	Hemolysis (%)
Complete hemolysis (+ve control)		1.509 ± 0.009	100
Isotonic solution (−ve control)			0
TiO_2_–Zn NC	1000	0.031 ± 0.004	0.3
	800	0.031 ± 0.006	0.6
	600	0.021 ± 0.002	0.7
	400	0.018 ± 0.002	0.6
	200	0.010 ± 0.001	0.4
	100	0.007 ± 0.002	0.4
	50	0.005 ± 0.002	0.3

### Comparative antioxidant performance of the TiO_2_–Zn nanocomposite

The TiO_2_–Zn NC exhibited concentration-dependent scavenging activity against DPPH radicals, with an IC_50_ value of 11.97 ± 0.04 µg mL^−1^ compared to 3.08 ± 0.02 µg mL^−1^ for ascorbic acid. At lower concentrations (1.9–31.2 µg mL^−1^), the scavenging percentages ranged from 29.5% to 60.7%, showing moderate activity. The difference between the nanocomposite and ascorbic acid narrowed at higher concentrations, reaching 90.8% and 94.6% at 500 µg mL^−1^ and 94.8% and 97.8% at 1000 µg mL^−1^, respectively (Table 3). The nanocomposite's scavenging percentage increased by 65.3 percentage points across the concentration range, demonstrating its capacity to neutralize DPPH radicals, though with lower efficiency than the reference compound.

**Table 3:** DPPH and ABTS radical scavenging activity of the TiO_2_–Zn NC and ascorbic acid at varying concentrations

Conc. (µg mL^−1^)	Antioxidant scavenging activity			
	TiO_2_–Zn NC DPPH scavenging % IC_50_ = 11.97 ± 0.04 µg mL^−1^	Ascorbic acid DPPH scavenging % IC_50_ = 3.08 ± 0.02 µg mL^−1^	TiO_2_–Zn NC ABTS˙^+^ scavenging% IC_50_ = 7.65 ± 0.09 µg mL^−1^	ABTS˙^+^ ascorbic acid scavenging % IC_50_ = 4.29 ± 0.09 µg mL^−1^
1.9	29.5	42.7	35.9	44.5
3.9	37.5	49.5	42.3	50.4
7.8	46	57.9	50.6	54.5
15.6	52.1	66.2	55.8	58.2
31.2	60.7	74.1	64.5	66.3
62.5	68.8	82.6	72.4	74.2
125	76.2	90	79.1	80.6
250	84.9	92.6	85.5	87.5
500	90.8	94.6	90.6	94.4
1000	94.8	97.8	93.7	96.1

For ABTS˙^+^ radical scavenging activity, the nanocomposite showed better performance in the ABTS assay, exhibiting an IC_50_ of 7.65 ± 0.09 µg mL^−1^ compared to 4.29 ± 0.09 µg mL^−1^ for ascorbic acid. The scavenging percentage ranged from 35.9% at the lowest concentration to 93.7% at 1000 µg mL^−1^, a 57.8 percentage-point increase. The nanocomposite's values maintained proximity to ascorbic acid values throughout the concentration series, with differences of 8.6% at 1.9 µg mL^−1^ and just 2.4% at the maximum concentration. Between 62.5 and 250 µg mL^−1^, the nanocomposite achieved 72.4–85.5% scavenging, indicating substantial radical neutralization within the practical concentration range (Table 3). Based on the IC_50_ values obtained, particularly 7.65 µg mL^−1^ for ABTS, the TiO_2_–Zn NC showed considerable potential as an antioxidant agent and was worthy of further investigation for applications requiring free radical scavenging properties.

TiO_2_–Zn NCs neutralize DPPH and ABTS radicals by donating electrons through single-electron transfer pathways, where ABTS reacts *via* sequential proton loss electron transfer (SPLET) mechanisms in water, while DPPH follows similar routes in alcoholic media.^49^ At the interface between TiO_2_ and ZnO crystals, heterojunction structures permit excited electrons to move from ZnO's conduction band into TiO_2_'s conduction band, and holes simultaneously migrate in reverse, from TiO_2_'s valence band to ZnO's valence band,^50^ which suppresses electron–hole recombination and extends the lifespan of reactive oxygen species involved in scavenging free radicals.^51^

Green synthesis using biological sources (bacteria, plants, and algae) offers distinct advantages by depositing bioactive metabolites, including proteins, polysaccharides, and secondary metabolites, onto nanoparticle surfaces during formation.^7^ Such metabolites serve dual functions as stabilizing agents and direct radical scavengers that amplify the antioxidant capacity beyond what chemical synthesis achieves.^52^ For instance, *Mucor racemosus*-mediated ZnO NPs demonstrated an IC_50_ value of 69.2 µg mL^−1^, with inhibition reaching 68.36% at a 200 µg mL^−1^ concentration,^53^ while lemon extract-mediated Zn–TiO_2_ nanocomposites achieved 94% DPPH scavenging at a 50 µL concentration compared to 91% for standard antioxidants,^44^ and plant-synthesized TiO_2_ NPs exhibited IC_50_ values ranging from 48.66 to 109.94 µg mL^−1^ across DPPH, ABTS, and H_2_O_2_ assays.^54^

The biosynthetic route, therefore, not only provides ecofriendly production but also functionalizes nanocomposite surfaces with organic ligands that directly participate in electron donation to radicals, creating a synergistic effect between inorganic electron transfer mechanisms and organic radical scavenging.^55^

### Anti-inflammatory profile of the TiO_2_–Zn NC

The biogenic TiO_2_–Zn NC exhibited significant anti-inflammatory activity, with dose-dependent inhibition of both COX-1 and COX-2. Against COX-2, the NC achieved an IC_50_ of 14.13 ± 0.5 µg mL^−1^, demonstrating stronger selectivity than its COX-1 inhibition (IC_50_ = 25.91 ± 0.3 µg mL^−1^), yielding a selectivity ratio of approximately 1.8-fold in favor of COX-2. Notable inhibition milestones included 49.3% COX-2 inhibition at just 7.8 µg mL^−1^ and 77.3% inhibition at 250 µg mL^−1^, while at the maximum concentration of 1000 µg mL^−1^, the NC exhibited 88.5% COX-1 inhibition and 90.6% COX-2 inhibition (Table 4). The NC demonstrated a promising COX-2 preferential profile, which is therapeutically desirable because COX-2 mediates inflammatory responses, while COX-1 serves protective gastrointestinal functions.

**Table 4:** *In vitro* COX-1 and COX-2 inhibitory activity of the biogenic TiO_2_–Zn NC

Conc. (µg mL^−1^)	COX inhibition assessment			
	TiO_2_–Zn NC COX-1 inhibition % IC_50_ = 25.91 ± 0.3 µg mL^−1^	Celecoxib COX-1 inhibition % IC_50_ = 3.42 ± 0.9 µg mL^−1^	TiO_2_–Zn NC COX-2 inhibition % IC_50_ = 14.13 ± 0.5 µg mL^−1^	Celecoxib COX-2 inhibition % IC_50_ = 4.11 ± 0.5 µg mL^−1^
0.5	9	29.3	15.3	27.8
1	16.4	38.7	26.5	31.5
2	21.1	45.4	31.3	47.3
3.9	29.4	50.9	37.5	51.4
7.8	37.3	58	49.3	57.5
15.6	46.5	64.8	51.4	62.9
31.25	52.1	72.5	57.5	69.8
62.5	58.4	78	62.1	76.7
125	65.3	86	69.2	84.3
250	73.7	90.3	77.3	89.1
500	82.8	94.1	83.2	92.8
1000	88.5	98	90.6	97.3

The inhibition patterns showed concentration-dependent increase across the entire range tested (0.5–1000 µg mL^−1^), with substantial activity emerging above 15.6 µg mL^−1^, where both enzymes showed greater than 50% inhibition, positioning these biogenic nanocomposites as viable candidates for anti-inflammatory applications with reduced gastrointestinal side effects compared to nonselective NSAIDs.

Regarding the BSA assessment, the NC demonstrated considerable anti-inflammatory activity by inhibiting protein denaturation, with an IC_50_ of 2.78 ± 0.05 µg mL^−1^. The NC displayed concentration-dependent protection against BSA denaturation across the tested range of 1.56–200 µg mL^−1^, with 50.8% inhibition at 3.125 µg mL^−1^, which increased to 89.3% at 100 µg mL^−1^ and 93.4% at the maximum concentration of 200 µg mL^−1^ (Table 5). The low IC_50_ value indicates a strong membrane-stabilizing capacity, as protein denaturation is a key mechanism underlying inflammation, where heat-induced unfolding mimics pathological conditions. At 25 µg mL^−1^, the NC achieved 78.3% inhibition, showing substantial activity well below cytotoxic thresholds. Compared to diclofenac sodium (IC_50_ = 1.63 ± 0.02 µg mL^−1^), the NC exhibited approximately 1.7-fold higher IC_50_ values, yet both materials exhibited comparable maximum inhibition percentages above 90% at higher concentrations.

**Table 5:** Protein denaturation inhibition of the TiO_2_–Zn NC and diclofenac sodium using the BSA assay

Conc. (µg mL^−1^)	BSA inhibition analysis	
	TiO_2_–Zn NC inhibition % IC_50_ = 2.78 ± 0.05 µg mL^−1^	Diclofenac sodium inhibition % IC_50_ = 1.63 ± 0.02 µg mL^−1^
1.56	40.7	45.5
3.12	50.8	55.8
6.25	58.7	65.0
12.5	68.8	75.2
25	78.3	82.8
50	83.9	90.2
100	89.3	92.5
200	93.4	96.2

Research has demonstrated that TiO_2_-NPs trigger COX-2 expression in human periodontal ligament cells by generating reactive oxygen species (ROS), a process mediated through NF-κB signalling activation.^56^ Interestingly, the incorporation of ZnO NPs into TiO_2_ results in diminished cytotoxic and genotoxic effects, attributed to TiO_2_'s capacity to adsorb Zn^2+^ ions, thereby altering inflammatory pathways through antagonistic mechanisms.^57^ Studies on ZnO NPs integrated within TiO_2_ nanotubes have revealed substantial anti-inflammatory effects by suppressing both macrophage proliferation and adhesion, indirectly suggesting decreased COX-2 activity, because this enzyme plays a central role in inflammatory cascades.^58^ The selective nature of TiO_2_–Zn NCs becomes apparent in their preferential inhibition of COX-2, which experiences upregulation during inflammatory states, and their minimal effect on COX-1, thereby circumventing adverse effects commonly linked to nonselective NSAIDs.^59^ Beyond enzyme inhibition, these nanocomposites markedly decrease proinflammatory cytokine levels, including IL-6 and inducible nitric oxide synthase (iNOS), within macrophages, signifying potent anti-inflammatory action.^60^ The synergistic architecture of TiO_2_–Zn NCs thus provides multiple therapeutic avenues through targeted COX-2 suppression, cytokine regulation, and macrophage function modulation, while TiO_2_'s adsorption of Zn^2+^ ions concurrently mitigates cytotoxic consequences and influences COX-2-related biological pathways.^61^

### Antimicrobial evaluation of the biogenic TiO_2_–Zn NC

**Fig. 8:**
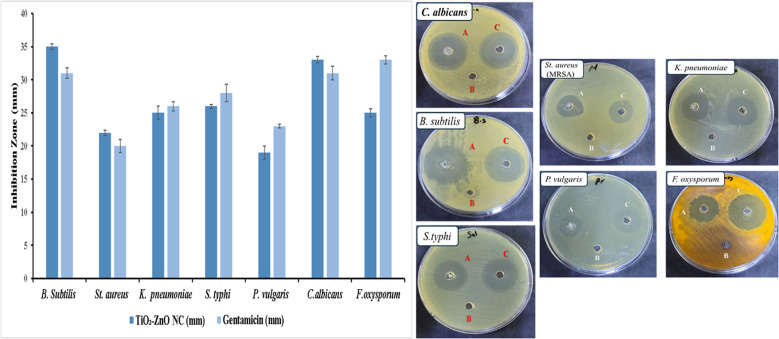
Antimicrobial activity of the TiO_2_–ZnO NC (A) and gentamicin (C) against bacterial and fungal pathogens compared to the blank control (B), measured by the inhibition zone diameter (mm). Fluconazole was used as the reference antifungal drug against fungal pathogens.

The biogenic TiO_2_–Zn NC exhibited an inhibition zone of 35 ± 0.4 mm against *B. subtilis*, compared to 31 ± 0.8 mm exhibited by gentamicin. At the same time, the nanocomposite and gentamicin exhibited inhibition zones against methicillin-resistant *S. aureus* (MRSA) of 22 ± 0.4 mm and 20 ± 1.0 mm, respectively, indicating superior activity against Gram-positive bacteria. Among this group, *B. subtilis* showed the largest inhibition zone, while MRSA showed the smallest. For Gram-negative bacteria, the nanocomposite and gentamicin exhibited inhibition zones of 25 ± 1.0 mm *vs.* 26 ± 0.7 mm for *K. pneumoniae*, 26 ± 0.3 mm *vs.* 28 ± 1.3 mm for *S. typhi*, and 19 ± 1.0 mm *vs.* 23 ± 0.3 mm for *P. vulgaris*, respectively (Fig. 8), revealing reduced effectiveness against Gram-negative bacteria. *P. vulgaris* showed the weakest inhibition at 19 ± 1.0 mm, while *S. typhi* exhibited the strongest at 26 ± 0.3 mm within this category (Fig. 8).

Among fungal pathogens, *C. albicans* showed an inhibition zone of 33 ± 0.5 mm for the nanocomposite *vs.* 31 ± 1.0 mm for fluconazole, while *F. oxysporum* showed 25 ± 0.6 mm compared to fluconazole's 33 ± 0.6 mm (Fig. 8), where the nanocomposite matched or exceeded fluconazole against the yeast but underperformed against the filamentous fungus. The nanocomposite demonstrated the strongest antimicrobial potential against *B. subtilis* (35 ± 0.4 mm) and *C. albicans* (33 ± 0.5 mm), while *P. vulgaris* proved most resistant (19 ± 1.0 mm, Fig. 8), suggesting selective efficacy influenced by the cell wall architecture and metabolic pathways unique to each microbial species.

### Antidiabetic potential of the TiO_2_–Zn NC through α-amylase and α-glucosidase inhibition

The biogenic NC demonstrated inhibitory activity against α-amylase, with an IC_50_ of 12.98 ± 0.88 µg mL^−1^, compared with 7.31 ± 0.11 µg mL^−1^ for acarbose. At the initial concentration of 1.95 µg mL^−1^, the NC achieved 26.7% inhibition, which increased progressively to 95.8% at 1000 µg mL^−1^, a total increment of 69.1 percentage points. The inhibition percentages remained within 6–8% of acarbose values across most concentrations, with the difference narrowing to just 1.4% at the highest dose (95.8% *vs.* 97.2%, Table 6). Between 31.25 and 250 µg mL^−1^, the nanocomposite exhibited 62.8–81.4% inhibition, covering the therapeutically relevant concentration range.

**Table 6:** α-Amylase and α-glucosidase inhibitory activity of the TiO_2_–Zn NC compared to the acarbose standard at varying concentrations

*In vitro* antidiabetic assessment				
Conc. (µg mL^−1^)	α-Amylase inhibition		α-Glucosidase inhibition	
	TiO_2_–Zn NC inhibition % IC_50_ = 12.98 ± 0.88 µg mL^−1^	Acarbose inhibition % IC_50_ = 7.31 ± 0.11 µg mL^−1^	TiO_2_–Zn NC inhibition % IC_50_ = 9.34 ± 0.32 µg mL^−1^	Acarbose inhibition % IC_50_ = 5.02 ± 0.19 µg mL^−1^
1.95	26.7	32.9	32	38.7
3.9	35.7	42.1	39.5	47.1
7.81	44.8	51	48	55.2
15.62	55.1	59.6	56.3	61.2
31.25	62.8	66.5	65.1	67.5
62.5	70.1	75.1	71.4	75.5
125	76	82.3	77.6	82.8
250	81.4	89.6	83.7	89.1
500	89.6	92	89.6	93.6
1000	95.8	97.2	93	96.7

The relatively close IC_50_ values and consistent inhibition pattern across the dose range indicate that the nanocomposite effectively blocks α-amylase activity, though requiring modestly higher concentrations than the standard drug.

Regarding α-glucosidase inhibition, the nanocomposite showed stronger activity against α-glucosidase, with an IC_50_ of 9.34 ± 0.32 µg mL^−1^, compared to 5.02 ± 0.19 µg mL^−1^ for acarbose. Inhibition values ranged from 32% at 1.95 µg mL^−1^ to 93% at 1000 µg mL^−1^, representing a 61 percentage-point increase. The NC maintained inhibition percentages of 5–7% below acarbose levels throughout the low-to-mid concentration range, with this gap reducing to 3.7% at the maximum concentration. At clinically relevant concentrations (62.5–250 µg mL^−1^), inhibition ranged from 71.4% to 83.7%, demonstrating substantial enzyme blockade (Table 6). The IC_50_ value below 10 µg mL^−1^, combined with inhibition exceeding 89% at higher concentrations, positions the TiO_2_–Zn NC as a candidate material for glucose management applications targeting postprandial hyperglycemia.

According to our findings, *Cydonia oblonga*-mediated ZnO NPs (20–50 nm, spherical) inhibited α-amylase by 81.7% and α-glucosidase by 86.9% at 100 µg mL^−1^.^62^ Furthermore, ZnO NPs from *Myristica fragrans* (spherical/elliptical, 41–23 nm) exhibited α-amylase and α-glucosidase IC_50_ values of 73.23 ± 0.42 and 65.21 ± 0.49 µg mL^−1^, respectively.^63^ Cube-shaped ZnO NPs from *Lessertia montana* inhibited α-amylase and α-glucosidase at IC_50_ concentrations of 0.120 and 0.037 g L^−1^, respectively.^64^*Streptomyces vinaceusdrappus*-mediated TiO_2_ NPs (spherical, 10–50 nm, anatase) demonstrated IC_50_ values of 69.3 µg mL^−1^ against α-amylase and 40.81 µg mL^−1^ against α-glucosidase.^27^ TiO_2_ NPs enhanced α-amylase production in *Aspergillus niger*, raising the specific activity from 12 037 to 15 523 U mg^−1^.^65^ Another study reported that the immobilization of α-amylase onto TiO_2_ NPs preserved 95% of the enzymatic activity and improved heat resistance, with nearly complete activity recovery following thermal deactivation.^66^ Another research showed that TiO_2_ NPs reduced salivary α-amylase function by 34% under *in vitro* conditions, though this inhibitory effect weakened in intestinal environments.^67^

TiO_2_–Zn NCs demonstrate antidiabetic efficacy through several integrated biological pathways. These nanostructures function by competitively blocking α-amylase through active site occupation, thereby restricting starch accessibility, whereas α-glucosidase experiences noncompetitive suppression *via* allosteric site binding, which modifies the enzyme structure, ultimately delaying carbohydrate breakdown and regulating postmeal blood glucose elevation.^68^ The gradual liberation of Zn^2+^ ions proves instrumental in facilitating insulin biosynthesis, storage, and release from pancreatic β-cells, alongside amplifying insulin responsiveness by stimulating glucose transporter protein expression (GLUT-2 and GLUT-4) and activating glucokinase, a pivotal enzyme governing glucose metabolism.^69,70^

Beyond enzyme modulation, ZnO nanoparticles trigger GLUT-4 membrane translocation, accelerate β-cell regeneration, and diminish oxidative burden, consequently preserving pancreatic islet architectural integrity.^71^ The therapeutic profile expands through the inhibition of AGE formation, addressing critical diabetic sequelae such as neurodegeneration, obesity, renal impairment, and retinopathy.^72^ This integrated strategy, encompassing enzyme suppression, insulin pathway enhancement, β-cell preservation, and oxidative stress mitigation, establishes TiO_2_–Zn NCs as viable therapeutic alternatives with reduced gastrointestinal complications relative to standard medications, including acarbose, miglitol, and voglibose.^73,74^

This study presents initial evidence that biogenic TiO_2_–Zn NCs synthesized *via Bacillus tequilensis* MYG163 metabolites possess multiple therapeutic properties. Future work should prioritize animal models of wound healing and hyperglycemia, followed by mechanistic studies employing gene expression profiling. Investigating photocatalytic performance under visible light could expand environmental applications, while scale-up feasibility and long-term colloidal stability testing would address industrial viability. Examining the nanocomposite's behavior in complex biological matrices and against drug-resistant clinical isolates would better define its therapeutic boundaries and inform rational formulation strategies.

## Conclusion

The TiO_2_–Zn NC biosynthesized through *Bacillus tequilensis* MYG163 metabolites exhibited multifunctional biomedical properties across five therapeutic domains. The spherical nanoparticles (primary size = 8–15 nm, hydrodynamic diameter = 87.3 nm) demonstrated structural integrity through anatase–wurtzite phase coexistence and maintained colloidal stability at a −34.5 mV zeta potential. Hemolytic activity stayed well below safety thresholds, confirming blood compatibility. The nanocomposite scavenged DPPH and ABTS radicals with concentration-dependent efficiency. Anti-inflammatory tests revealed preferential COX-2 suppression over COX-1 and significant inhibition of protein denaturation. Antimicrobial assays showed strong activity against Gram-positive bacteria and fungi, surpassing standard agents, though G −ve strains exhibited moderate resistance. Antidiabetic screening demonstrated dual enzyme blockade, with stronger α-glucosidase inhibition than α-amylase suppression.

The convergence of these activities within a single bacterial-mediated synthesis platform indicates that Red Sea-derived *Bacillus* strains can generate metal oxide heterojunctions capable of addressing interconnected pathological processes. Bacterial metabolites functioned simultaneously as reducing agents, crystal growth directors, and surface stabilizers, yielding nanostructures with therapeutic attributes that chemical synthesis routes rarely achieve. These results establish a foundation for developing multitarget therapeutic agents that operate through complementary biochemical pathways rather than single-mechanism interventions.

## Author contributions

Methodology: I. M. I., M. A., D. A., N. M. Z., F. A. S. A., I. A. Investigation: H. A. A., Z. A., A. E. A. Formal analysis and visualization: A. G., A. E. A., Z. A., I. A., D. A., F. A. S. A. Data curation and validation: D. A., M. A., I. M. I., A. A. A. Writing – original draft: A. G., H. A. A., D. A. Writing – review & editing: A. G., I. A., Z. A.

## Conflicts of interest

The authors state that they have no conflicts of interest to declare.

## Data Availability

Data are available by contacting the corresponding author.
